# Supplementary material for: Complexome profiling of the Chlamydomonas psb28 mutant reveals TEF5 as an early PSII assembly factor
Source: Plant Cell. 2025 Mar 17;37(6):koaf055. doi: 10.1093/plcell/koaf055 (PMC12164590; doi:10.1093/plcell/koaf055)
Supplement: koaf055_Supplementary_Data [file koaf055_supplementary_data.zip › psb28_interactive.zip!/plots/intensity/qualityPlots/peptides/_Cre16.g659700.t1.2.html]

### Cre16.g659700.t1.2

Trivial name:   
SeqCoverage [median]: 14.70  
Mass: 10.66 kDa  
  
 

| Sequence | MissedCleavage | Mass [Da] | wtIntensity | muIntensity | Score |
| --- | --- | --- | --- | --- | --- |
| ASKVVAAATAPATKK | 2 | 1412.84 | 0.00 | 1100.46 | 100.11 |
| VVAAATAPATK | 0 | 998.58 | 4052.57 | 15258.70 | 108.98 |
| VVAAATAPATKK | 1 | 1126.67 | 32704.25 | 42851.72 | 165.63 |
|  |  |  | 36756.8 | 59210.9 |  |
